# The incorporation of MALDI mass spectrometry imaging in studies to identify markers of toxicity following *in utero* opioid exposures in mouse fetuses

**DOI:** 10.3389/ftox.2024.1452974

**Published:** 2024-12-03

**Authors:** Dustyn Barnette, Amy L. Inselman, Pravin Kaldhone, Grace S. Lee, Kelly Davis, Sumit Sarkar, Pritpal Malhi, J. Edward Fisher, Joseph P. Hanig, Richard D. Beger, E. Ellen Jones

**Affiliations:** ^1^ National Center for Toxicological Research (FDA), Division of Systems Biology, Jefferson, AR, United States; ^2^ Center for Drug Evaluation and Research (CDER), Office of Testing and Research, Silver Spring, MD, United States; ^3^ National Center for Toxicological Research (FDA), Toxicologic Pathology Associates, Jefferson, AR, United States; ^4^ National Center for Toxicological Research (FDA), Division of Neurotoxicology, Jefferson, AR, United States; ^5^ Center for Drug Evaluation and Research (CDER), Division of Pharmacology Toxicology for Neuroscience, Silver Spring, MD, United States

**Keywords:** morphine, exencephaly, opioid, MALDI MSI, lipids, hypoxia, toxicology, development

## Abstract

**Introduction:**

In 2015, the FDA released a Drug Safety Communication regarding a possible link between opioid exposure during early pregnancy and an increased risk of fetal neural tube defects (NTDs). At the time, the indications for opioid use during pregnancy were not changed due to incomplete maternal toxicity data and limitations in human and animal studies. To assess these knowledge gaps, largescale animal studies are ongoing; however, state-of-the-art technologies have emerged as promising tools to assess otherwise non-standard endpoints. Matrix-assisted laser desorption/ionization mass spectrometry imaging (MALDI MSI) is a dynamic approach capable of generating 2D ion images to visualize the distribution of an analyte of interest across a tissue section.

**Methods:**

Given the importance of lipid metabolism and neurotransmitters in the developing central nervous system, this study incorporates MALDI MSI to assess lipid distributions across mouse gestational day (GD) 18 fetuses, with and without observable NTDs following maternal exposure on GD 8 to morphine (400 mg/kg BW) or the NTD positive control valproic acid (VPA) (500 mg/kg BW).

**Results:**

Analysis of whole-body mouse fetuses revealed differential lipid distributions localized mainly in the brain and spinal cord, which included several phosphatidylcholine (PC) species such as PCs 34:1, 34:0, and 36:2 localized to the cortex or hippocampus and lyso PC 16:0 across all brain regions. Overall, differential lipids increased in with maternal morphine and VPA exposure. Neurotransmitter distributions across the brain using FMP-10 derivatizing agent were also assessed, revealing morphine-specific changes.

**Discussion:**

The observed differential glycerophospholipid distributions in relation to treatment and NTD development in mouse fetuses provide potential targets for further investigation of molecular mechanisms of opioid-related developmental effects. Overall, these findings support the feasibility of incorporating MALDI MSI to assess non-standard endpoints of opioid exposure during gestation.

## 1 Introduction

Since 1999, more than 280,000 Americans have died from an opioid drug overdose. In 2021, 75% of drug overdose deaths, totaling more than 80,000, involved an opioid (CDC et al., 2023), representing a 4% increase from the previous year, which clearly depicts the devastating opioid crisis currently plaguing the United States. Unfortunately, opioid misuse among pregnant women is on the rise as well, which raises concerns regarding the devastating effects these drugs may have on the developing fetus (White, et al., 2022; Broussard, et al., 2011). For example, maternal opioid use has been reported to be associated with a 2-fold increase in neural tube defects (NTDs), such as exencephaly, a condition in which the brain and skull are not fully formed (Yazdy, et al., 2013). In response to these findings, the FDA issued a Drug Safety Communication in 2015 urging careful consideration when taking any pain medicine during pregnancy, including opioids (FDA, 2015); however, due to incomplete maternal toxicity data and limited animal and human studies, the FDA did not make new recommendations for opioid use during pregnancy. Nevertheless, there remains a critical need for further research to comprehensively assess the risks associated with fetal exposure to opioids. A previous study of morphine administered to pregnant mice showed that exposed litters had a statistically significant increase in the incidence of exencephaly, a precursor to anencephaly, yet the molecular mechanism behind the link between the opioid exposure and the NTD was not explored (Harpel and Gautieri, 1968; Morphine sulfate tablets, 2021). To address these knowledge gaps, large-scale clinical and animal studies at the FDA are ongoing. In addition to many of the endpoints traditionally assessed in a reproductive and developmental toxicity study, researchers are also assessing the incorporation of novel cutting-edge technologies into existing studies to identify otherwise nonstandard endpoints such as lipids and neurotransmitters.

Matrix-assisted laser desorption/ionization mass spectrometry imaging (MALDI MSI) is a dynamic, state-of-the-art approach capable of generating 2D ion images in which the distribution of an analyte of interest can be visualized across a tissue section. This approach is robust, label-free, and detects a wide range of analytes (Caprioli, et al., 1997; Aichler and Walch, 2015) including proteins (Stauber, et al., 2010), peptides (Minerva, et al., 2008; Debois, et al., 2010), *N-linked* glycans (Powers, et al., 2013), lipids (Fernandez, et al., 2011), small molecule drugs (Nilsson, et al., 2010), and metabolites (Khatib-Shahidi, et al., 2006). Correlation of MALDI MSI analyte distributions with tissue histopathology or immunohistochemistry on the same or adjacent tissue section(s) provides an in-depth assessment of the location of an analyte relative to the underlying tissue microenvironment (Balluff, et al., 2011). The incorporation of high resolution instruments, such as the Fourier-transform ion cyclotron resonance mass spectrometer (FTICR MS), within imaging workflows has increased the specificity, sensitivity, and spatial resolution achievable with this approach (Good, et al., 2022; Nilsson, et al., 2010; Shariatgorji, et al., 2019), propelling mass spectrometry-based imaging to the foreground of imaging modalities and further establishing it as a promising tool to investigate in-depth spatial molecular questions.

Although the field of MALDI MSI continues to grow expeditiously across all disciplines, there are only few reported studies using the approach in the field of reproductive and developmental toxicology, and far fewer using it to assess opioid exposures during pregnancy (Sarikahya, et al., 2022). Given the importance of lipid and neurotransmitter metabolism in brain and neutral tube development, the goal of this study was to assess the feasibility of incorporating MALDI MSI into existing workflows through an applied investigation of whether the tissue distributions of these analytes were impacted by morphine exposure. Using high resolution MALDI MSI, along with histopathology, morphine-specific lipid and neurotransmitter changes could be identified in the brains and spinal cords of whole-body mouse fetuses, further supporting the incorporation of these workflows in future reproductive toxicology studies.

## 2 Methods

### 2.1 Test articles and materials

Morphine sulfate (catalog number M1167; lot 1IG0879) was obtained from Spectrum Chemical Mfg. Corp. (New Brunswick, NJ, United States). Methanol, acetonitrile, and optimal cutting temperature (OCT) compound were purchased from Fisher Scientific (Pittsburgh, PA, United States). Valproic acid sodium salt (VPA; catalog number P4543; lot MKCJ7640) and sodium chloride (0.9% solution; catalog number S8776), acetone, 2,5 -dihydroxybenzoic acid (DHB) matrix, trifluoroacetic acid (TFA), reserpine internal standard, Fluro-Jade C (FJC), and DPX mounting medium were purchased from Millipore-Sigma (St. Louis, MO, United States). Indium tin oxide (ITO) slides for MALDI and ESI Tuning Mix were purchased from Bruker Daltonics (Bilerica, MA, United States). Microscope slides for pathology were purchased from Thermo Scientific (Waltham, MA, United States). Derivatizing agent 4-(anthracene-9-yl)-2-fluoro-1-methylpyridin-1-ium iodide (FMP-10) was purchased from Tag-ON AB (Uppsala, Sweden).

### 2.2 Dose and test article preparation

A total of three treatment groups were analyzed by MALDI MSI. VPA was selected as a positive control compound for the study with known links to NTDs (Hughes, et al., 2018). Doses were based on the results from a preliminary dose-range finding study in which the compounds induced NTDs (data will be reported elsewhere). Morphine was administered at 400 mg/kg BW, and VPA at 500 mg/kg BW. A vehicle group (0.9% sodium chloride) was also included for comparison.

The test articles were prepared in a 0.9% sodium chloride solution, filter sterilized (0.22 µm), stored in sealed plastic tubes at 4°C, and protected from light until use. All test article solutions were used within 3 days of preparation.

### 2.3 Animal source, maintenance, and in-life data collection

The study was conducted in an Association for Assessment and Accreditation of Laboratory Animal Care (AAALAC)-accredited facility. All animal procedures were approved by the NCTR Institutional Animal Care and Use Committee (IACUC) and followed the Guidelines set forth by the National Research Council’s Guide for the Care and Use of Laboratory Animals (Council National Research, 2011). Animals were conditioned to a 12-h light/12-h dark cycle with animal rooms maintained at 23°C (+3°C) with a relative humidity of 50% (+20%). Throughout the study, timed-pregnant animals were individually housed in standard microisolator top cages with Alpha-dri^®^ bedding material. Feed (NIH-07) and Millipore-filtered tap water (Jefferson, AR municipal supply) were provided *ad libitum*.

A total of 140 timed-pregnant CF-1 mice (strain code: 023) were purchased from Charles River Laboratories (Wilmington, MA, United States) and arrived at NCTR on gestational day (GD) 4 (day of vaginal plug detection designated as GD 0). On arrival, animals were assigned to treatment groups (20 animals per group) by a weight-ranked randomization procedure to ensure the mean initial weight of each treatment group was equivalent.

### 2.4 Animal dosing and collection of GD 18 fetuses

**FIGURE 1:**
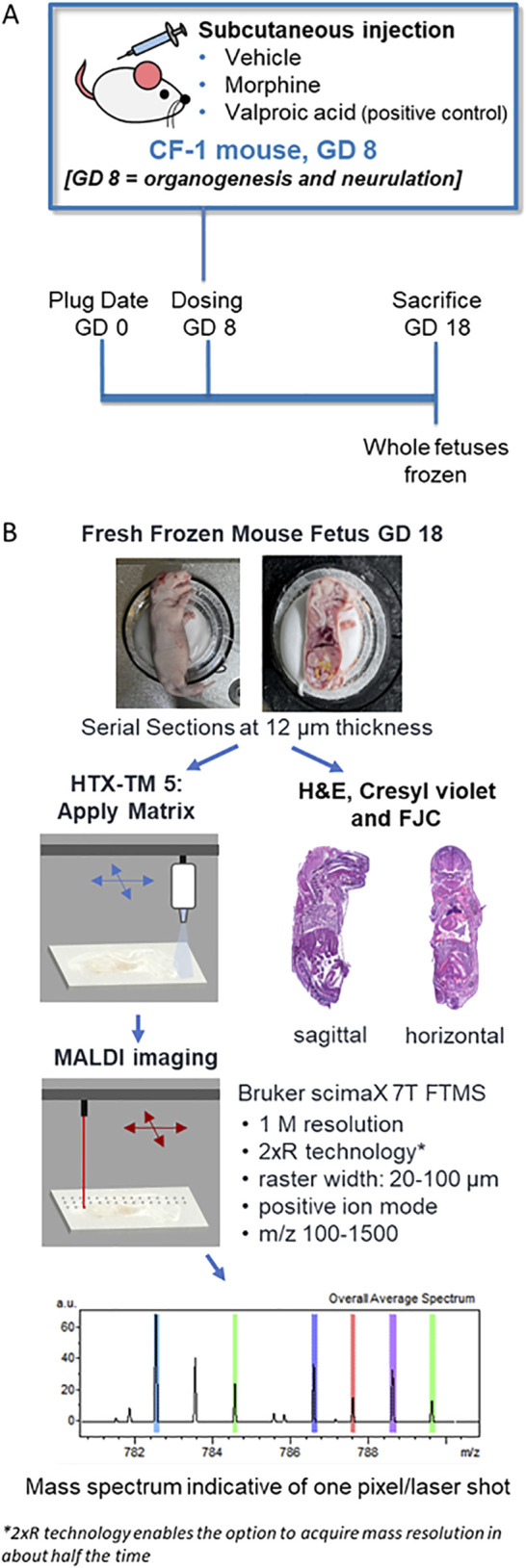
Experiment and methods overview. **(A)** Schematic of the *in vivo* experimental design for assessing morphine, valproic acid, and vehicle exposures during pregnancy using mouse models. **(B)** Processing methods overview of frozen mouse fetuses for MALDI MSI and histopathology staining. GD, gestational day; FJC, Fluoro-Jade C; FTMS, fourier transform mass spectrometry, H&E; hematoxylin and eosin.

An overview of the *in vivo* experimental design is outlined in Figure 1A. On GD 8 of pregnancy, timed-pregnant females were given a single dose of 0.9% saline (vehicle), morphine (400 mg/kg BW), or VPA (500 mg/kg BW) by subcutaneous injection. On GD 18, animals were euthanized for teratological assessment of fetuses (data not included) and collection for MALDI MSI imaging. The gravid uterus was removed from the dam, weighed, and opened for examination of individual fetuses for external gross malformations. Upon removal from the uterus, fetuses were euthanized.

Five litters were randomly selected from each treatment group for evaluation by MALDI MSI, and two fetuses from each of the selected litters were collected. If a litter contained a fetus with an external malformation (e.g., exencephaly), then the fetus with the malformation was collected alongside a fetus with no visible malformation. Fetuses were flash frozen on dry ice at the time of collection and stored at −80°C until sectioning.

### 2.5 Fetal sectioning and tissue preparation

Fresh frozen whole-body mouse fetuses were collected, sectioned, and analyzed as outlined in Figure 1B. Sagittal or horizontal sections were collected using a cryomicrotome (THM525 NX, Thermo Scientific Waltham, MA, United States). After test sectioning and scanning of some fetuses, fetuses selected for final MALDI MSI evaluation included one negative control fetus from a vehicle-treated dam, and three fetuses each from the 400 mg/kg BW morphine and 500 mg/kg BW VPA groups. Fetuses selected from the morphine and VPA groups consisted of two with no visible malformations and one with exencephaly. For the groups with no visible malformations, one fetus was used for sagittal sectioning, and the other fetus was used for horizontal sectioning. A single fetus from the vehicle group was used for both sagittal and horizontal sectioning; therefore, horizontal sections for that treatment group were all sectioned from half of a fetus previously cut along the midsagittal plane. Only sagittal sections were collected from the fetuses with exencephaly. A minimal amount of OCT was used to secure the fetuses for sectioning at −20°C. For sagittal sectioning, approximately forty sections at 100 µm were trimmed and discarded from the left side of the fetus until the brain and most of the spinal cord were visible, indicating the approximate midsagittal region of the mouse fetus. From this region, 12 µm thickness sections were cut and collected on a slide. Horizontal sections were collected that intersected the fetal brain at an angle between that of a traditional horizontal section and a coronal section, due to the angle of the head with respect to the rest of the body in the frozen samples. Collecting whole body horizontal sections from two regions of the fetus enabled the areas of interest that were identified from the sagittal images to be captured. The approximate region of the brain containing the thalamus/hypothalamus was determined to be about 6 mm from the tip of the fetus’s nose based on the examination of a series of full-body horizontal sections acquired from extra fetuses by a histopathologist. The region was reached by trimming and discarding approximately sixty horizontal sections at 100 µm each (6 mm total); then 12 µm sections were cut and collected for mounting for MALDI, hematoxylin and eosin (H&E), and Cresyl violet staining, while 10 µm sections were collected for FJC analysis. All sectioning was carefully discussed with a board-certified veterinary pathologist.

Sections for MALDI MSI were thaw-mounted onto indium tin oxide-coated glass slides, and then sprayed with matrix depending on the target analytes (see below). Sections for pathology were mounted on microscope slides and allowed to air dry at room temperature for 20–40 min, then fixed using various methods dependent on the stain. Slides for H&E were fixed by submerging in a 1:1 mixture of cold methanol and acetonitrile for 30 min. Slides for Cresyl violet staining were fixed in a 1:1 mixture of cold methanol and acetone for 30 min. Slides for FJC were prepared as described below. All slides were air dried following solvent submersion before being stained with either H&E or Cresyl violet to aid in the identification of brain subsites from MALDI-imaged serial sections.

### 2.6 FTICR MALDI MSI analysis for lipids

Mounted sections for lipid analysis were sprayed with DHB acid matrix at a concentration of 40 mg/mL in 70% methanol and 0.1% TFA using a M5 HTX Tm-sprayer (HTX Technologies, Chapel Hill, NC, United States) with a 0.18 mL/min flow rate, 1,200 mm/min velocity, and N2 pressure of 10 psi. The spraying nozzle was heated to 75°C and sprayed with eight passes using a tracking space of 1.5 mm and a 10 s drying time. All tissues were imaged using a Bruker ScimaX 7T FTICR mass spectrometer (Bruker Daltonics, Bremen, Germany) equipped with a dual ESI-MALDI (electrospray ionization MALDI source) and collected using 1 MW time-domain (0.1049 s transient length). The instrument was calibrated using ESI Tuning Mix (Agilent Technologies, Santa Clara, CA, United States) for mass accuracy to less than 1 ppm. Initial imaging runs were acquired with the sagittal sections in positive ionization mode using ftmsControl Version 2.3.0 covering *m/z* of 150–1,500 with a 1.0 s time of flight, 200 laser shots per pixel, a 100 µm raster width, and a resolution of 138,000 at *m/z* 622. The whole fetal body was scanned, and the laser was tuned using a reserpine standard (*m/z* 609.281) that was spiked into the matrix solution at a concentration of 2.5 μg/mL. The brains in horizontal sections were targeted with high resolution scans, which followed the same parameters described above, but with a 30 µm raster width, to assess lipid changes in specific brain regions.

Images were visualized using FlexImaging Version 5.0 with normalization by total ion count, and intensity scales were manually adjusted for each lipid adduct of interest. Data analysis was conducted using SCiLS Lab Version 2023b (RRID:SCR_014426), in which entire brains and specific regions were defined based on overlaid Cresyl violet stained serial sections. Initial screening of lipid peaks was conducted using the sagittal fetal sections (Mohammadi, et al., 2016), in which the top 200 peaks were identified in the whole brain area using a feature finding tool. The feature list was manually screened to identified those with parent masses that correspond to known lipid adducts based on literature reference for highly abundant lipids in the mouse brain. Peak identities were confirmed using the LIPIDMAPS database to match peaks to lipids based on mass accuracy with <1 ppm mass error tolerance.

Collision induced dissociation (CID) of some lipid peaks was conducted for further confirmation, using an RF frequency of 2 MHz, a collision RF amplitude of 1,200 Vpp, and collision energies of 10–20 V. The SCiLS receiver operating characteristic (ROC) analysis tool was used to assess the discrimination quality of lipid features in whole sagittal brain sections based on individual spectra (pixels) in single fetal specimens between control (group 1) and treatment (group 2) samples. This analysis also served as a metric based on area under the ROC curve (AUC) for selecting which lipids from the sagittal analysis (AUC <0.2 or AUC >0.8) to target in a more in-dept analysis of lipid changes within individual brain regions (cortex, hippocampus, thalamus, and hypothalamus) in the horizontal brain sections. Discriminating lipids were visualized by boxplots based on intensity values plotted for each pixel.

### 2.7 FTICR MALDI MSI analysis for labeled neurotransmitters

Horizontal mouse fetal sections for neurotransmitter analysis were collected as described above, but at 6.5 mm from the fetal nose to intersect with the substantia nigra. Sections were sprayed with FMP-10 reactive matrix (Tag-ON, Uppsala, Sweden) to tag and image neurotransmitter distributions in the brain by adapting a method described by others (Shariatgorji, et al., 2019). Briefly, mounted 12 µm thickness sections were sprayed with FMP-10 matrix at 1.8 mg/mL in 70% acetonitrile using a M5 HTX Tm-sprayer. FMP-10 matrix was applied with a 0.15 mL/min flow rate, 1,200 mm/min velocity, and N2 pressure of 10 psi. The spraying nozzle was heated to 90°C and sprayed with 30 passes and a tracking space of 1.5 mm.

The fetal sections were imaged using the Bruker ScimaX instrument described above in positive ionization mode. Only the brain regions were selected for scanning. Imaging runs were acquired using ftmsControl Version 2.3.0, covering m/z of 150–1,500 with a 0.6 s time of flight, 200 laser shots per pixel, a 50 µm raster width, and a resolution of 133,000 at *m/z* 698. Peaks representing four neurotransmitters were analyzed and identified based on reported m/z values (Shariatgorji, et al., 2019) with a mass accuracy of <1 ppm mass error tolerance.

### 2.8 FJC staining

A modified FJC staining protocol was followed (Schmued, et al., 1997). Briefly, horizontal sections (10 µm) were immediately processed for FJC staining after mounting on slides. Sections were dried on a slide warmer for 20–30 min. Dried slides were placed in 10% neutral buffered formalin for 15 min and washed twice in 0.1 M phosphate buffer for about 1 min each to remove formalin. Slides were placed in basic alcohol for 2 min, rinsed in 70% alcohol for 2 min, and then rinsed in Millipore filtered water for 1 min. Subsequently, the slides were incubated in 0.06% potassium permanganate solution for 2 min and washed in Millipore filtered water for 1 min. Slides were then placed in FJC for 10 min, followed by washing twice with Millipore filtered water for 2 min each and drying on a slide warmer at 60°C for about 5 min. Dried slides were cleared in xylene for 5–8 s and then coverslipped with DPX mounting medium. Once slides had dried at room temperature, the brains were examined with a microscope and the hippocampus regions were imaged at 10X and 20X.

## 3 Results

**FIGURE 2:**
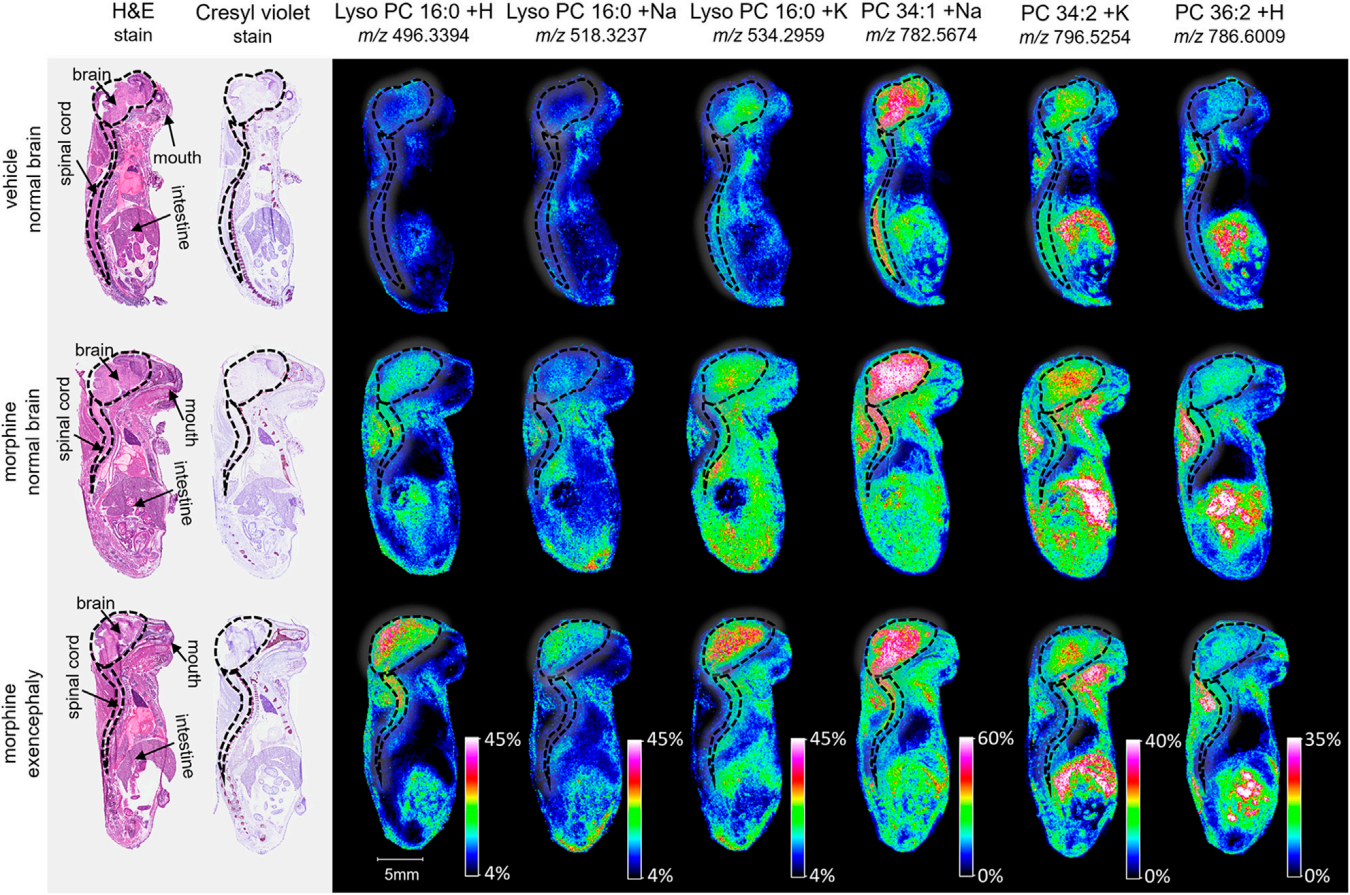
Imaging and lipid assessment of whole sagittal sections of high dose morphine-exposed mouse fetuses. Sagittal sections (12 µm) were taken from three whole body mouse fetuses for MALDI imaging: One exposed to vehicle control with normal brain development, one exposed to morphine (400 mg/kg BW) with no visible malformations, and one exposed to morphine with an exencephaly NTD. SCiLS ROC analysis identified six lipid adducts with discriminating qualities (AUC <0.2 or AUC >0.8), including lyso PC 16:0 + H, lyso PC 16:0 + H, lyso PC 16:0 + K, PC 34:1 +Na, PC 34:2 + K, and PC 36:2 + H. FlexImaging heatmaps show distributions and relative level intensities for targeted lipids normalized by total ion count. Dotted lines outline the brain and spinal cord.

GD 18 mouse fetuses were collected from pregnant dams which had been dosed on GD 8 with a single administration of either vehicle, morphine (400 mg/kg BW), or VPA (500 mg/kg BW) (Figure 1). Upon collection, all fetuses were examined for the presence or absence of NTDs (e.g., exencephaly). At least one instance of fetal exencephaly was observed in morphine and VPA treatment groups as confirmed by a veterinary pathologist (Figure 2A).

To investigate lipid distributions following morphine exposure, whole body mouse fetuses were analyzed. Vehicle control, morphine, or VPA-treated dams with normal brains or brains with exencephaly were sectioned sagittally and analyzed via MALDI MSI (one fetus for each treatment and brain development type). Although assessed, morphine and VPA could not be detected in the mouse fetuses, likely due to collection occurring 10 days post dose. MALDI MSI is capable of identifying hundreds of peaks within lipid imaging runs; however, for this study the top 200 peaks were identified using the SCiLS feature finding tool (Supplementary Table S1). Of these, 23 were identified as lipids and their appropriate adducts based on accurate mass measurements and selected for further analysis (Mohammadi, et al., 2016). The identified peaks represented sodium, potassium, and/or hydrogen adducts of 10 lipid species (Table 1).

**TABLE 1:** Lipids observed in MALDI images of mouse fetal sections.

Lipid	Adducts observed	Observed parent (*m/z*)	LIPID MAPS reported (*m/z*)	Mass accuracy (ppm)	CID fragment (*m/z*)
Lyso PC 16:0	+ H	496.3394	496.3398	−0.805899507	184.07
	+ Na	518.3219	518.3217	0.38586075	146.98
	+ K	534.2959	534.2956	0.561486937	162.96
PC 30:0	+ H	706.5442	706.5381	0.424605552	
PC 32:0	+ H	734.5700	734.5694	0.816805056	184.07
	+ Na	756.5508	756.5514	−0.793072354	146.98
	+ K	772.5250	772.5253	−0.388336796	162.95
PC 32:1	+ H	732.5600	732.5538	0.136508745	
	+ Na	754.5338	754.5357	−0.132531834	
	+ K	770.5193	770.5097	−0.648921097	
PC 34:0	+ H	762.6003	762.6007	−0.524520893	184.07
	+ Na	784.5829	784.5827	0.25491258	146.98
PC 34:1	+ H	760.5857	760.5851	0.788866361	184.07
	+ Na	782.5668	782.5670	−0.255569172	
	+ K	798.5403	798.5410	−0.876598697	162.95
PC 34:2	+ H	796.5254	796.5253	0.125545290	
PC 36:1	+ H	788.6159	788.6164	−0.634021813	184.07
	+ Na	810.5988	810.5983	0.616828335	146.98
	+ K	826.5718	826.5723	−0.604907762	162.95
PC 36:2	+ H	786.6009	786.6007	0.254258609	184.07
	+ Na	808.5830	808.5827	0.371019563	
	+ K	824.5565	824.5566	−0.121277302	162.95
PC 38:6	+ H	806.5766	806.5694	0.371945675	

**FIGURE 3:**
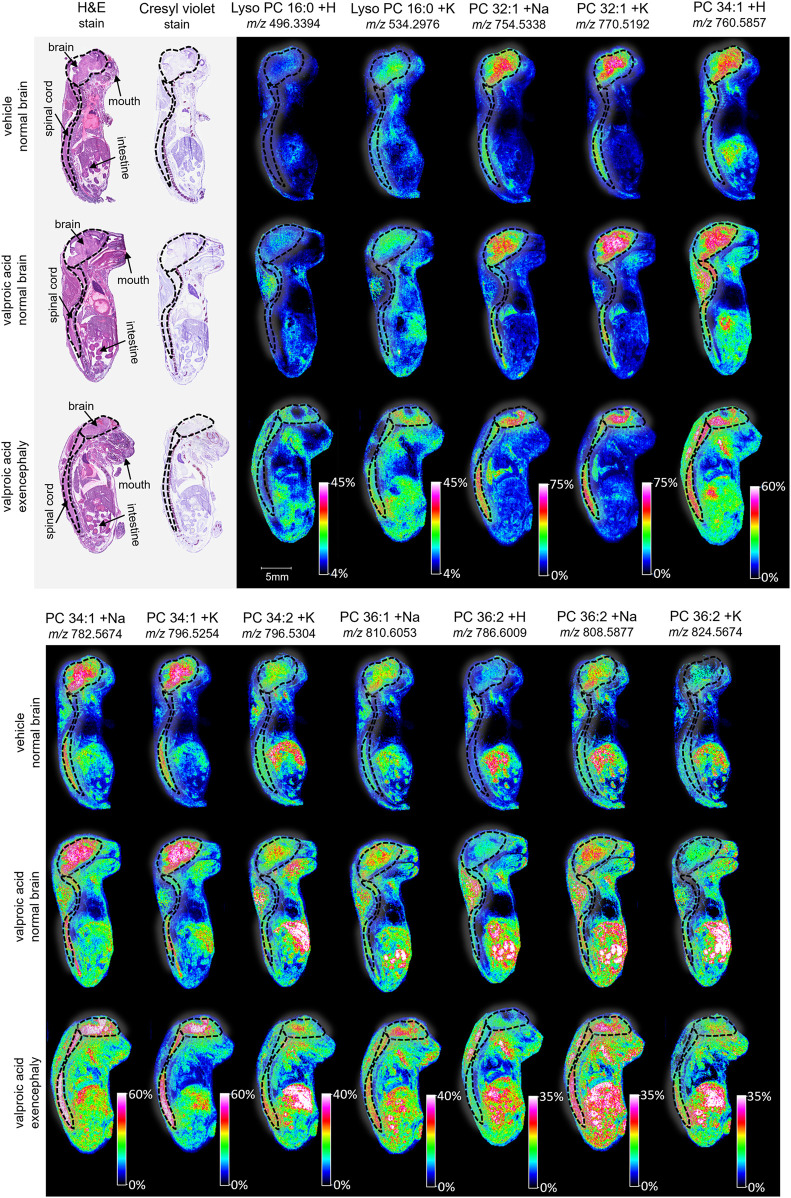
Imaging and lipid assessment of whole sagittal sections of high dose VPA-exposed mouse fetuses. Sagittal sections (12 µm) were taken from three whole body mouse fetuses for MALDI imaging: One exposed to vehicle control with normal brain development, one exposed to VPA (500 mg/kg BW) with no visible malformations, and one exposed to VPA with an exencephaly NTD. SCiLS ROC analysis identified twelve lipid adducts with discriminating qualities (AUC <0.2 or AUC >0.8), including lyso PC 16:0 + H, lyos PC 16:0 + K, PC 32:1 +Na, PC 32:1 + K, PC 34:1 + H, PC 34:1 +Na, PC 34:1 + K, PC 34:2 + K, PC 36:1 +Na, PC 36:2 + H, PC 36:2 +Na, and PC 36:2 + K. FlexImaging heatmaps show distributions and relative level intensities for targeted lipids normalized by total ion count. Dotted lines outline the brain and spinal cord.

MALDI imaging and ROC analyses of the 23 lipid adducts in the whole sagittal brain sections were conducted to compare non-exposed normal brains to both morphine-exposed normal and morphine-exposed exencephaly brains (Supplementary Table S2). ROC analysis identified many discriminating lipids (AUC <0.2 or AUC >0.8). In the normal brains, four lipids were differential between the control and the morphine-exposed fetal brain (Supplementary Table S2) including lyso PC 16:0 and PCs 32:0, 34:1, and 36:2 (Figure 2). In the exencephaly brain, only lyso PC 16:0 was differential. Boxplots of lipid intensities show that overall lipid distributions increased flowing morphine exposure morphine exposure (Supplementary Figure S1A). Meanwhile, three lipids were discriminating in the spinal cords for morphine-exposed fetuses (with normal or exencephaly brains) in the spinal cords, which were lyso PC 16:0, PC 34:1, and PC 34:2 (Supplementary Figure S1B). In MALDI images of VPA-exposed fetuses compared to controls (Figure 3), no discriminating lipids were identified in the normal brain. Two were identified in the exencephaly brains, which were lyso PC 16:0 and PC 36:2, though only one adduct of each was discriminating (Supplementary Table S3; Supplementary Figure S2A). In the spinal cords of VPA-exposed fetuses, ten lipid adducts representing six lipid species were discriminating, which included lyso PC 16:0, PC 32:1, PC 34:1, PC 34:2, PC 36:1, and PC 36:2. Of those, only lyso PC 16:0 and PC 36:2 were discriminating for the fetus with the normal brain. Overall lipid distributions increased in VPA-exposed fetuses (Supplementary Figure S2B). MALDI images of all of the remaining 23 lipid adducts not identified as discriminating are in supporting information (Supplementary Figure S3). Accurate mass measurements of parent compounds for all targeted lipids were within <1 ppm of values reported in LIPID MAPs (Table 1) (LIPID MAPS, 2023) and some were confirmed via CID fragmentation (Supplementary Figure S4). Characteristic CID fragments were detected at *m/z* 184.07 for protonated PC adducts, *m/z* 146.98 for sodiated adducts, and *m/z* 162.96 for PC potassium adducts (Table 1).

**FIGURE 4:**
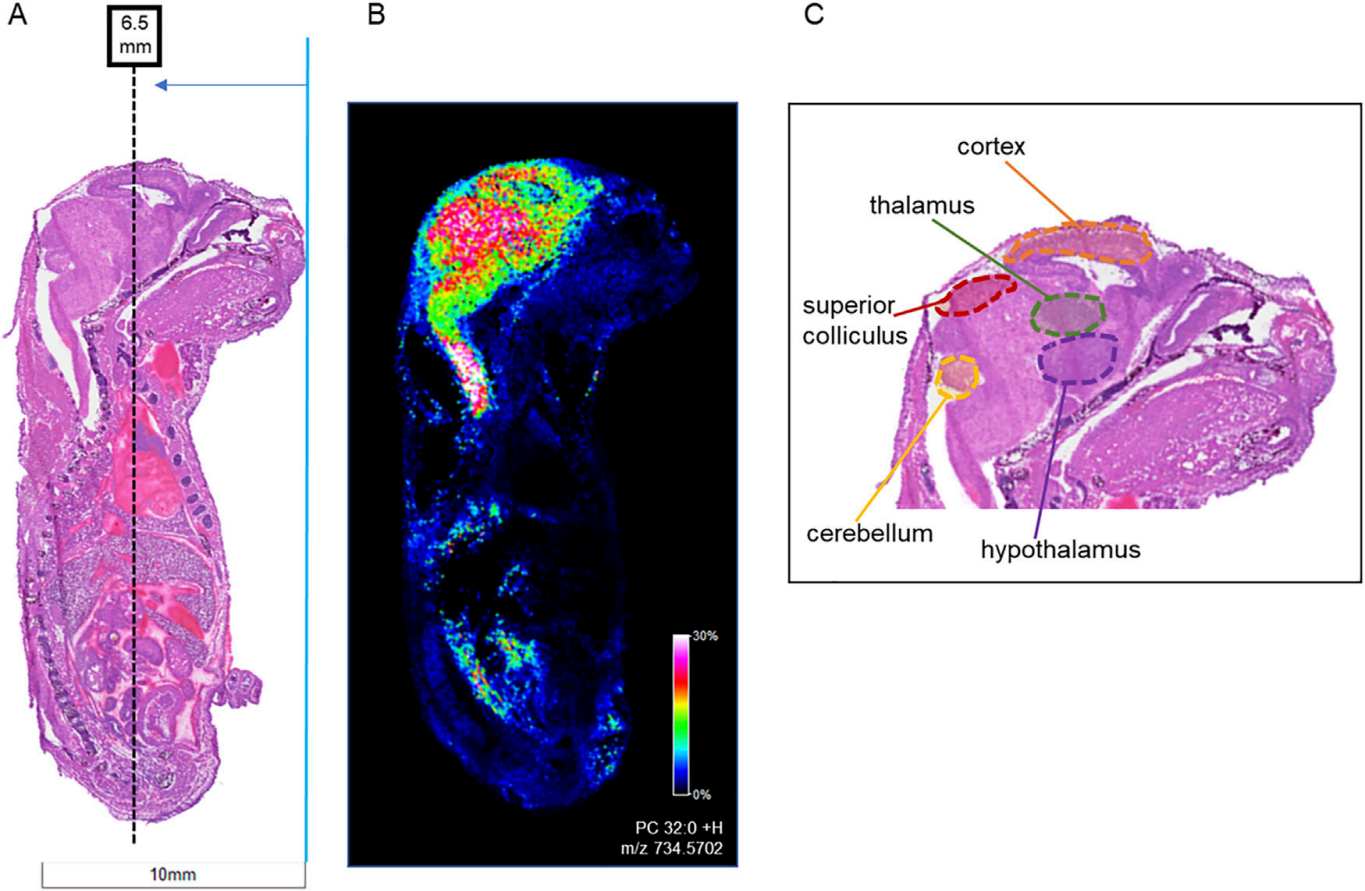
Locations of horizontal sections taken from mouse fetuses relative to sagittal sections. Horizontal sections (12 µm) were taken from whole body mouse fetuses exposed to vehicle control or to 400 mg/kg BW morphine. **(A)** H&E-stained sagittal section shows location of horizontal sections collected at ∼6.0 mm from the tip of the fetal nose in order to intersect areas of high lipid concentration as shown in **(B)** MALDI IMS sagittal section imaged for PC 32:0 (m/z 734.570). **(C)** The targeted region intersects with the cerebral cortex, thalamus, and hypothalamus of the brain.

**FIGURE 5:**
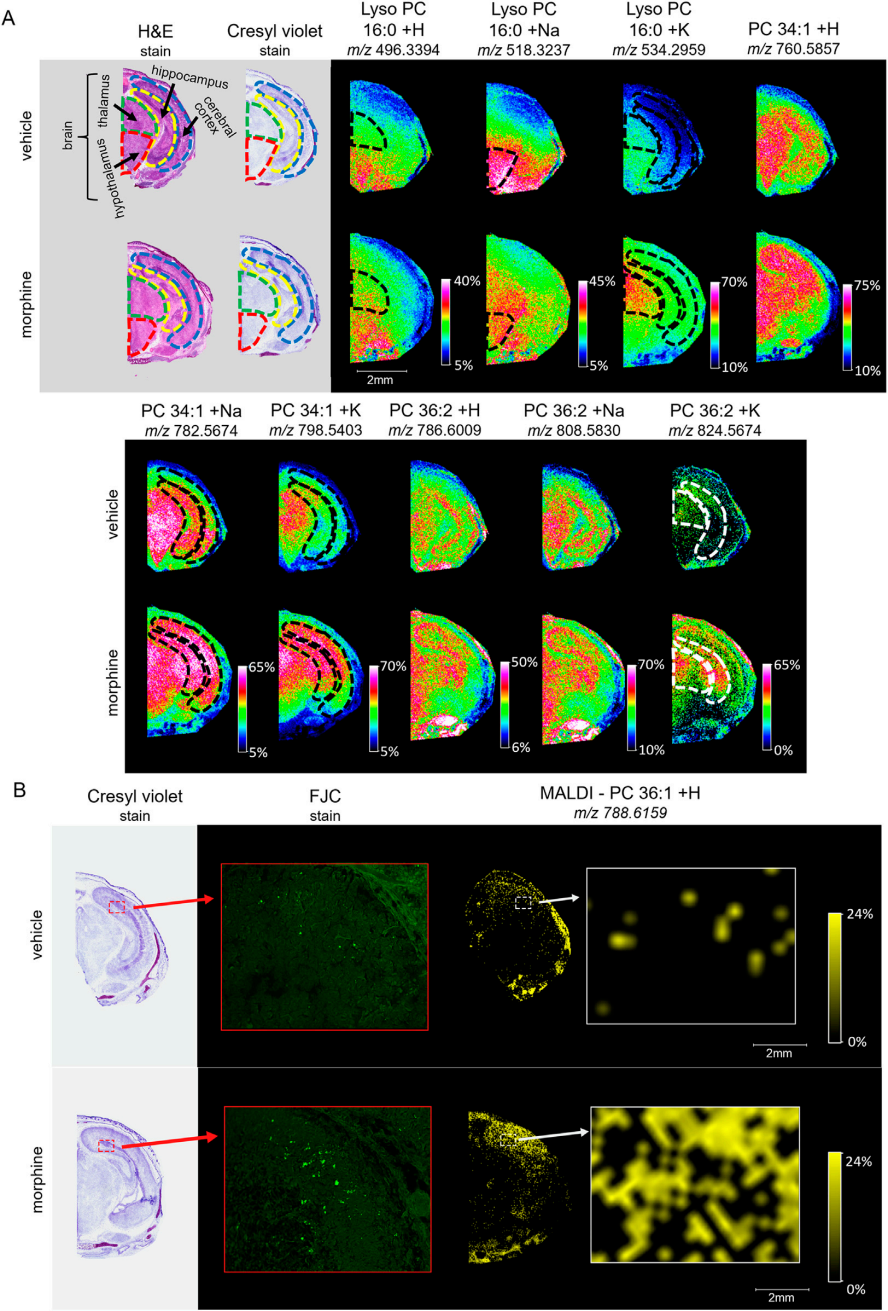
Imaging and staining of head horizontal sections of morphine-exposed mouse fetuses. The regions collected were measured at around 6.5 mm from the end of the fetuses’ noses. **(A)** Horizontal sections (12 µm) were taken from single mouse fetuses with normal brain development exposed to vehicle or morphine (400 mg/kg BW) for high resolution MALDI scans of the right half of the brain. FlexImaging heatmaps normalized by total ion count show 30 µm raster width scans for protonated, sodiated, and potassiated adducts of lyso PC 16:0, PC 34:1, and PC 36:2, and the brain regions are outlined for thalamus (green dashed outline), hypothalamus (red dashed outline), cerebral cortex (blue dashed outline), and hippocampus (yellow dashed outline), as determined based on stained serial sections. **(B)** Serial horizontal sections (10 µm) taken from mouse fetuses prenatally exposed to vehicle or to 400 mg/kg morphine were stained with Cresyl violet, and FJC. MALDI scans were taken at 30 µm raster width for PC 36:1 and normalized by total ion count. Arrows indicate magnification transitions, with the high-resolution images of the H&E− and Cresyl violet-stained fetal sections taken at a magnification of ×1.3 and the FJC images taken at ×20 magnification.

Following the SCiLS analysis, the three discriminating lipid species identified from the whole brain analysis in morphine-exposed mouse fetuses were assessed in the tissues using a higher resolution (30 µm raster width). To enable imaging of the specific brain regions of interest, a method was developed to section the fetuses horizontally (see Methods; Figure 4). Mouse fetuses with exencephaly were not included in the horizontal section analysis due to the size and developmental differences from the healthy brains, rendering them incomparable. High resolution MALDI MSI analysis of the horizontal brain sections was conducted as shown in Figure 5, Panel A. ROC analysis for specific brain regions revealed zero discriminating features when assessing the horizontal brain section as a whole; however, regional analyses of the cortex, hippocampus, hypothalamus, and thalamus revealed discriminating peaks for some lipid adducts in relation to morphine exposure (Table 2). Lyso PC 16:0 distribution is elevated in morphine treated brains across the entire brain. PC 34:1 is also elevated specifically in the cortex and the hippocampus. PC 36:2 distribution is increased in the thalamus and the hippocampus. Future statistical analysis with larger sample sizes will be required to determine biological significance for the lipid changes identified in the brain regions to make more accurate conclusions about treatment-dependent changes.

**TABLE 2:** ROC analysis results for lipid adduct peaks in MALDI scans of mouse fetal brain horizontal sections.

Llipids			Brain region (ROC *p*-values)^a^				
Species	Adduct	*m/z*	Whole brain	Thalamus	Hypothalamus	Hippocampus	Cortex
Lyso PC 16:0	+ H	496.3394	0.402	**0.171**	0.457	0.485	0.412
	+ Na	518.3219	0.480	0.366	**0.924**	0.423	0.419
	+ K	534.2959	0.256	**0.043**	0.727	**0.036**	**0.168**
PC 34:1	+ H	760.5857	0.347	0.352	0.480	0.248	0.241
	+ Na	782.5668	0.337	0.476	0.739	**0.109**	**0.117**
	+ K	798.5403	0.247	0.253	0.607	**0.030**	**0.114**
PC 36:2	+ H	786.6009	0.356	0.209	0.327	0.271	0.483
	+ Na	808.5830	0.343	0.265	0.545	0.221	0.286
	+ K	824.5565	0.261	**0.198**	0.398	**0.099**	0.331

^a^ ROC, analysis conducted using SCiLS, discriminating features tool for control (group 1) *versus* treatment specimen (group 2). Discriminating features were identified based on AUC <0.2 or AUC >0.8 (bolded).

To investigate whether morphine-exposure is associated with degenerating cells, horizontal fetal brain sections were stained with FJC, a non-discriminatory marker of neuronal degeneration. FJC positive cells were detected at higher levels in the morphine-exposed fetal brain, specifically in the hippocampus (Figure 5B). MALDI MSI images of serial horizontal sections scanned at a 30 µm raster width were compared with the FJC-stained sections in order to observe lipid distributions at sites of neural degeneration. Notably, the signal density for PC 36:1 increases in regions with higher incidences of FJC-positive structures, indicating a possible correlation between potential morphine-related increases in PC 36:1 lipids with sites of neurodegeneration in the hippocampus.

**FIGURE 6:**
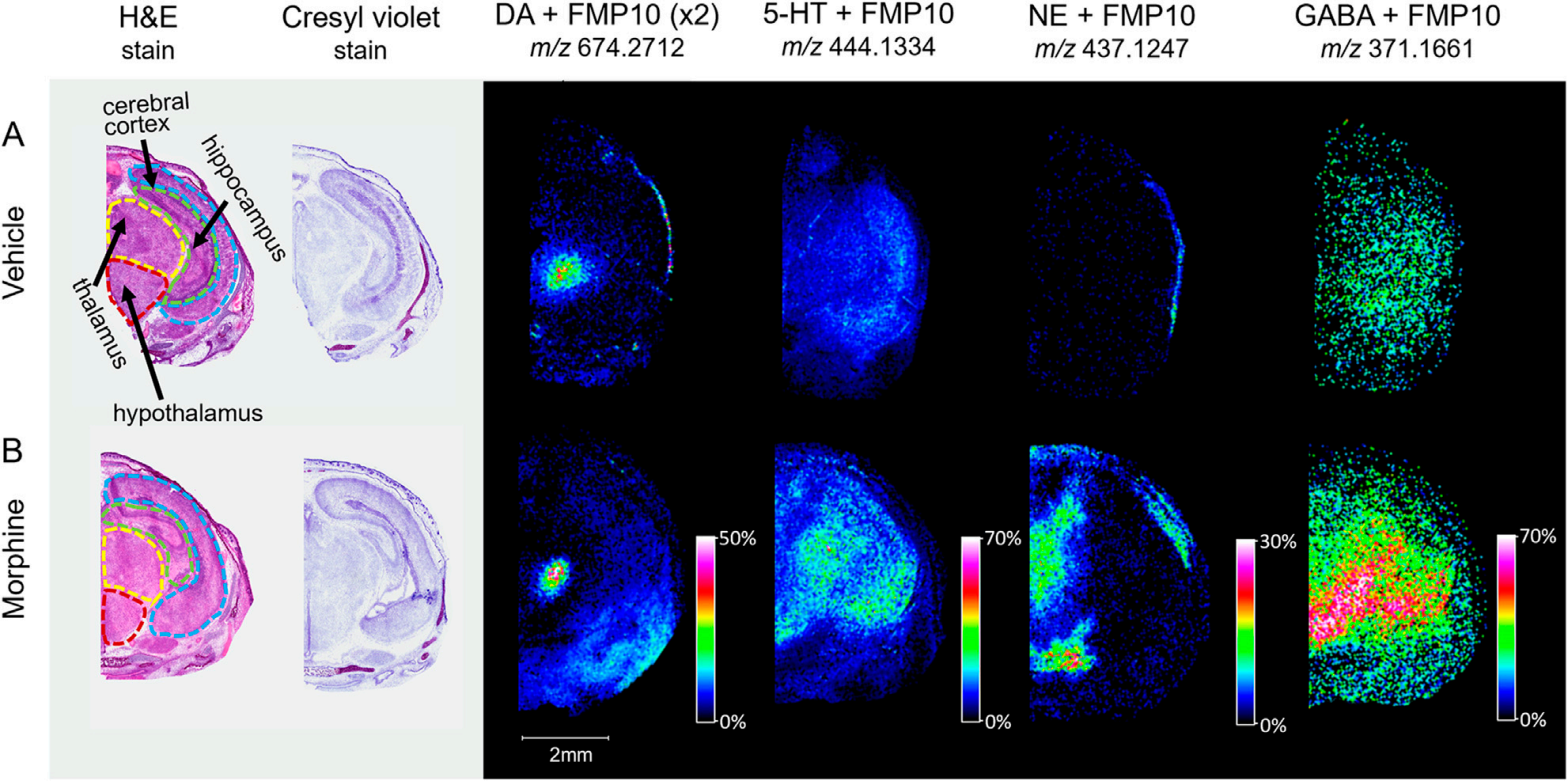
Imaging of tagged neurotransmitters in morphine-exposed mouse fetuses. Horizontal sections (12 µm) taken from mouse fetuses exposed to **(A)** vehicle control or **(B)** morphine (400 mg/kg BW) were sprayed with FMP-10 matrix for derivatization. The regions for section collection were measured at around 6.5 mm from the end of the fetus’s nose. Brain regions were selected for MALDI imaging with 50 µm raster width and normalized by total ion count.

With the potential impact of opioids on the brain and neurodegeneration, a newly available derivatizing agent (FMP-10) was used as a proof-of-concept approach for assessing neurotransmitter distribution (Shariatgorji, et al., 2019). Horizontal sections intersecting the substantia nigra of vehicle and morphine-exposed fetuses were tagged and imaged. Distributions of dopamine (DA), serotonin (5-HT), norepinephrine (NE), and γ-aminobutyric acid (GABA) neurotransmitters tagged with FMP-10 were identified (Figure 6) based on parent mass (Shariatgorji, et al., 2019), and distributions of NE and GABA are higher in the morphine-exposed fetuses.

## 4 Discussion

MALDI MSI is a cutting-edge tool to map specific analyte changes across a tissue section in relation to tissue pathology. In the field of reproductive and developmental toxicity, the use of this approach has the potential to measure non-standard endpoints such as lipids which may provide important clues concerning molecular mechanisms or pathways involved in development. This study utilized high resolution MALDI MSI to identify lipid changes in mouse fetuses following *in utero* morphine exposure, and the results highlight the advantages of incorporating this approach within future studies.

The overall study focus was on fetal development following maternal exposure to opioids during the period of organogenesis. Fetuses were exposed to morphine on GD 8, and whole fetuses were collected on GD 18 to assess developmental changes. A small subset of mouse fetuses was designated for MALDI MSI analysis of lipids in the animals, with a specific interest on brain and spinal cord development. The dosing strategies utilized are based on previous studies in which pregnant mice given morphine showed a statistically significant increase in the incidence of exencephaly in litters exposed to 200 mg/kg to 500 mg/kg on GD 8, and from 100 mg/kg to 500 mg/kg on GD 9 (Harpel and Gautieri, 1968; Morphine sulfate tablets, 2021). VPA has known links to NTD development, and so was used as a positive control in the study (Anon., 2011; Kao, et al., 1981; Padmanabhan and Mohamed Shafiullah, 2003; Okada, et al., 2016; Hughes, et al., 2018). The inability to detect parent drug using MALDI imaging 10 days after dosing was unsurprising given that the half-life in mice is 0.5 h for morphine (Gabel, et al., 2022; Gabel, et al., 2020; Kalvass, et al., 2007) and 0.8 h for VPA (Loscher, 1977; Methaneethorn, 2018; Zaccara et al., 1988). Future study designs that include more acute time points (e.g., corresponding to C_max_) and/or accumulation studies corresponding to different dosing regimens could be insightful to assess acute or chronic drug and metabolite distributions in relation to other analytes of interest, such as lipids and neurotransmitters.

Due to their involvement in membrane stability, signaling, and disease (Natesan and Kim, 2021; Yoon, et al., 2021; Mi, et al., 2023) lipids were assessed by MALDI MSI. They are a favorable class of analytes to assess using this approach given their abundance, ease of ionization, structure, and mass range (Djambazova, et al., 2023; Jones, et al., 2014; de la Monte, et al., 2018; Krishnan, et al., 2023; Rivera, et al., 2022; Xie, et al., 2022). Although MALDI MSI of whole-body mouse fetuses has proven successful in studies looking at proteins (Thomas, et al., 2013) and lipids (Berry, et al., 2011; Chaurand, et al., 2011), it has not been fully appreciated in the field of reproductive and developmental toxicology, and only a handful of studies have utilized the approach to assess markers of toxicity following drug exposure (Sarikahya, et al., 2022). Other mass spectrometry imaging platforms, such as DESI-MS (desorption electrospray mass spectrometry), have also been used to image metabolites and lipids (Leon, et al., 2019; Perez, et al., 2017), and could also be useful moving forward; however, most existing imaging studies have been limited to early embryonic development with a focus on single cell divisions (Goncalves, et al., 2014; Onjiko, et al., 2015; Onjiko, et al., 2016; Onjiko, et al., 2017; Laskowski, et al., 2017; Duenas, et al., 2017; Burnum, et al., 2009).

MALDI MSI successfully identified hundreds of peaks across the fetal tissue sections; however, in this study, 23 lipid adducts representing 10 lipid species were identified from a screen of the top 200 peaks. CID of target peaks confirmed their respective lipid identities. Protonated, potassiated, and sodiated PC adducts yielded fragments of *m/z* 184.07, 162.96 and 146.98, respectively, which are consistent with previous reports (Klein and Brodbelt, 2018; Hsu and Turk, 2003; Blank, et al., 2022; Murphy, et al., 2009). Lipids identified included lyso PC 16:0, which is associated with neurodegeneration (Law, et al., 2019); five PCs that may be linked to hypoxia (34:2, 34:1, 34:0, 36:2, and 36:1) (Jiang, et al., 2015), which has been associated with NTDs (Silvestro, et al., 2020; Chen, et al., 1999); and PC 32:0, which is a predominantly gray matter lipid in mouse brain tissue (Mohammadi, et al., 2016). Although hypoxia is one possible pathway through which lipid metabolism may be affected, further research would be needed to support this theory and rule out other possible molecular mechanisms which may alter lipid expression, including reactive oxygen species, folate metabolism and the Land’s cycle (Engel, et al., 2021; Tung and Winn, 2011; Sarikahya, et al., 2022; Steele, et al., 2020; Lands, 1958; Shindou and Shimizu, 2009).

An advantage of MALDI MSI within this study to more traditional approaches is the ability to map analyte changes to specific regions of the brain. Although lipid changes were consistent across individual regions of the whole brain, they were not uniform changes, which could be missed by routine LC-MS/MS methods. Horizontal sections of the mouse fetal brains allowed visualization of lipid distributions between the thalamus, hypothalamus, cerebral cortex, and hippocampus in a single section. Within these sections for the morphine-exposed fetus, lyso PC 16:0 was identified as a discriminating feature in all targeted brain regions, and other lipids had discriminating qualities only in specific regions, which were the hippocampus and cortex for PC 34:1 and the hippocampus and thalamus for PC 36:2. These region-specific changes were masked when the whole horizontal brain was assessed. Lipid changes in the hypothalamus and thalamus have been reported previously in DESI MSI studies of lipid distributions across adult rat brain regions with morphine exposure (Bodzon-Kulakowska, et al., 2017); however, only negatively charged lipids were assessed.

Quantitative MALDI MSI measurements, although possible, were not assessed in the current study, but coupling with LC-MS/MS on a larger number of animals will be imperative in future studies to rule out biological variability and establish statistical significance. Technical replicates will be necessary to rule out artifacts; however, the general consistency of lipid trends between the horizontal and sagittal sections is promising. Additionally, with the potential impact of opioids on the brain and neurodegeneration, evaluation of neurotransmitters, which are now detectable by MALDI MSI due to a newly available derivatizing agent (FMP-10) (Shariatgorji, et al., 2019), will be important for follow up studies. Imaging of tagged proof-of-concept horizontal sections intersecting the substantia nigra of vehicle and morphine-exposed fetuses was only conducted on a small subset of samples and is by no means comprehensive, yet the tissue distributions of the identified neurotransmitters are higher in the morphine-exposed fetuses, specifically norepinephrine and GABA. Work is ongoing to determine whether these changes are consistent and to determine the biological relevance, but this method represents a novel approach to assess neurotransmitters for use in developmental studies. The results of this study demonstrated that incorporation of MALDI MSI within existing studies is seamless within the context of a larger workflow to be utilized across multiple future studies. Additionally, these types of assessments could enhance understanding of the molecular mechanisms of toxicity for different types of opioids, such as fentanyl and heroin, which may have faster mechanisms of action and increased potentials for addiction.

## 5 Conclusion

Overall, these findings represent an innovative approach to assess toxicity in whole-body and brain sections of mouse fetuses following *in utero* exposure to opioids. The observed differential glycerophospholipid levels and distributions within fetal neural tissue, in relation to treatment and development of NTDs, provide potential molecular targets for further investigation and may offer clues regarding the possible molecular mechanisms of opioid-related developmental effects. This study also demonstrates the utility of MALDI MSI to assess non-standard endpoints, which could have broader applications in the field of reproductive and developmental toxicity and neurotoxicity.

## Data Availability

The raw data supporting the conclusions of this article will be made available by the authors, without undue reservation.
