# Supplementary material for: The incorporation of MALDI mass spectrometry imaging in studies to identify markers of toxicity following in utero opioid exposures in mouse fetuses
Source: Front Toxicol. 2024 Dec 3;6:1452974. doi: 10.3389/ftox.2024.1452974 (PMC11651024; doi:10.3389/ftox.2024.1452974)
Supplement: Supplementary file 1 [file Table1.docx]

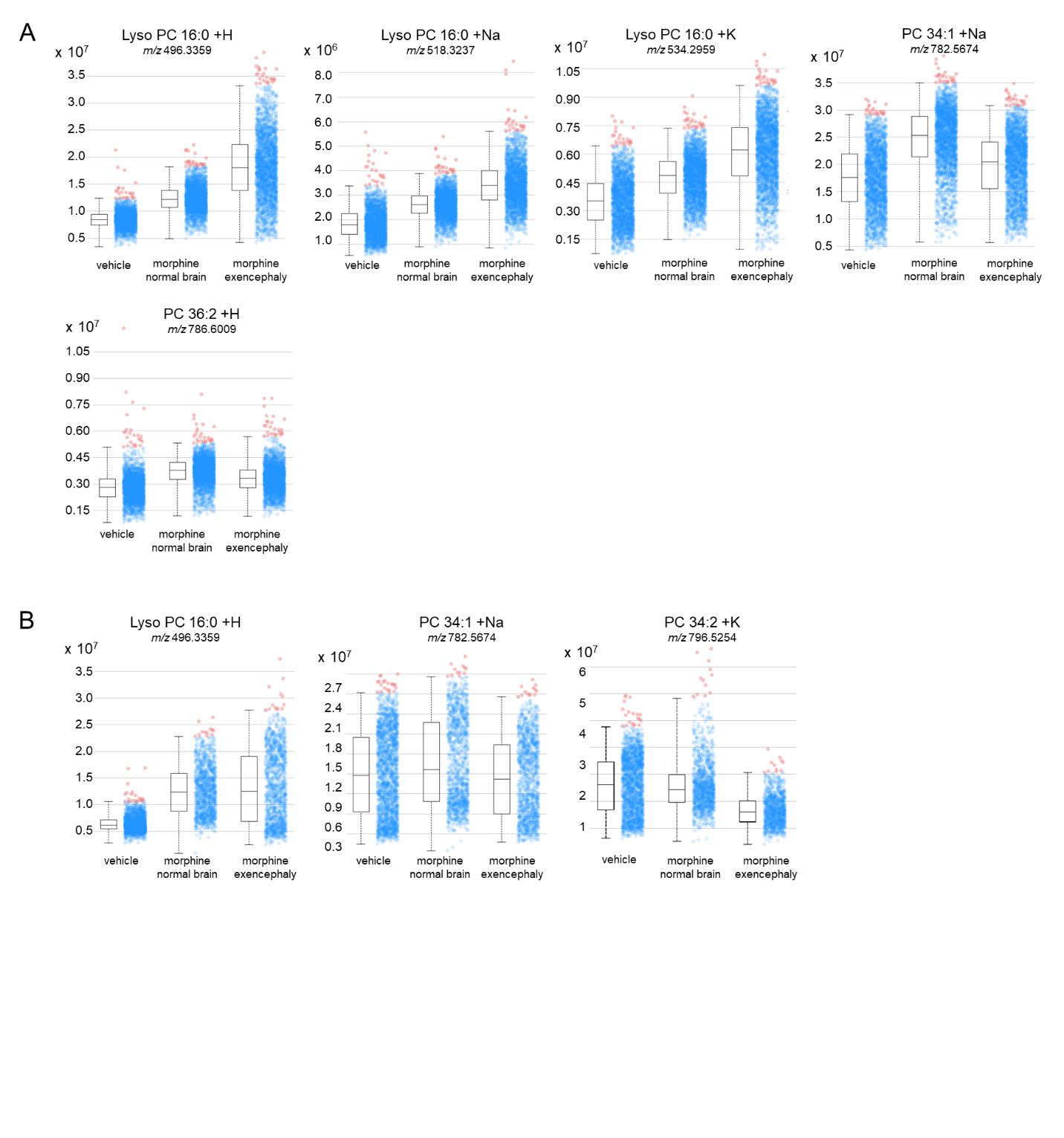


**Figure S1. Lipid peak intensities for MALDI scans of sagittal sections of morphine-exposed fetuses.** Boxplots show peak intensities from MALDI scans (Figure 2) of three whole body mouse fetus sagittal sections (12 µm): One exposed to vehicle control with normal brain development, one exposed to morphine (400 mg/kg BW) with no visible malformations, and one exposed to morphine with an exencephaly NTD. Lipids shown were identified as discriminating lipid peaks using SCiLS ROC analysis (AUC < 0.2 or AUC > 0.8) for **(A)** whole brains and **(B)** spinal cords. Each dot represents the signal intensity at a single pixel in the MALDI image, with red dots representing outliers (1.5X interquartile range).


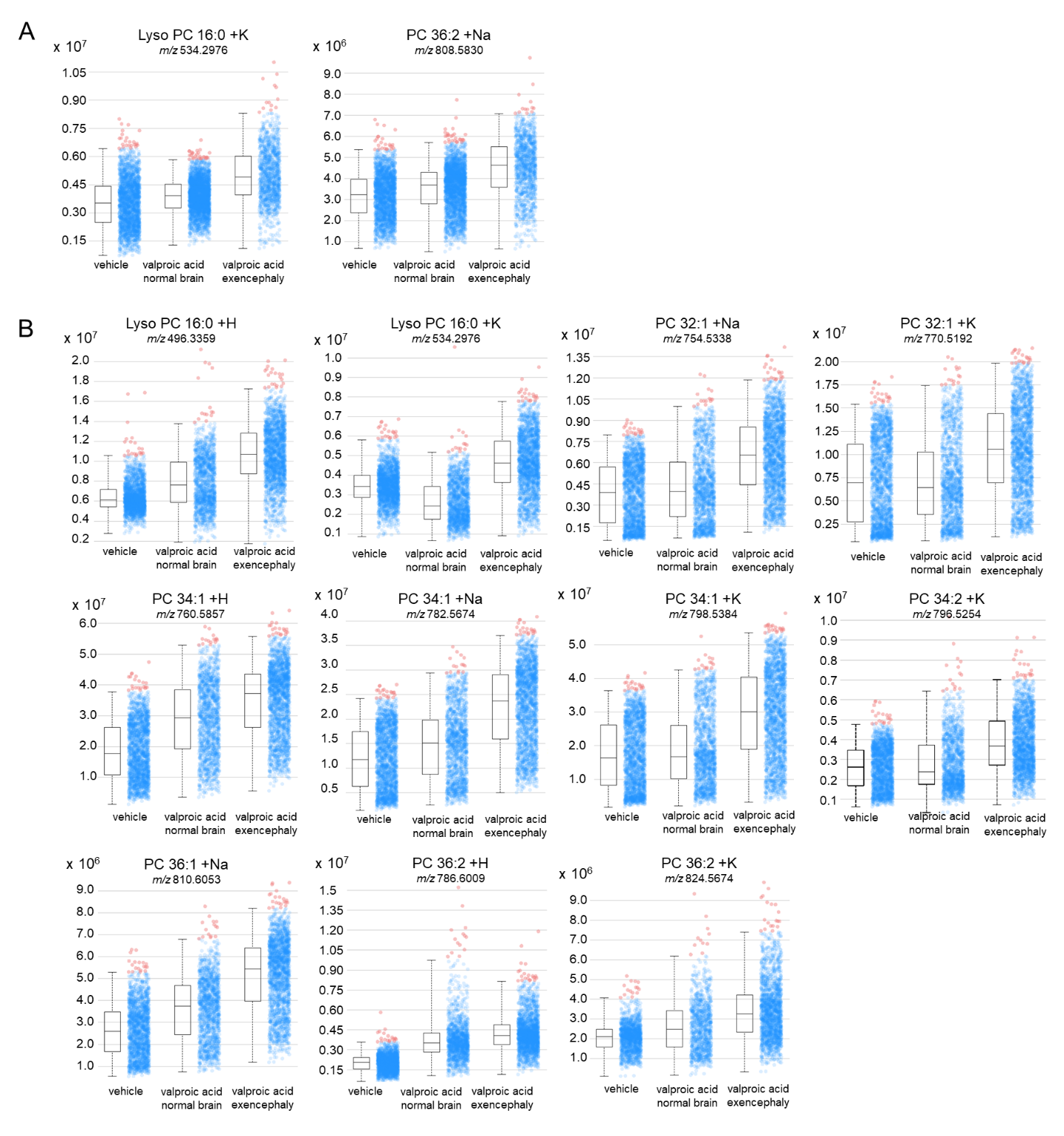


**Figure S2. Lipid peak intensities for MALDI scans of sagittal sections of VPA-exposed fetuses.** Boxplots show lipid intensities from MALDI scans (Figure 3) of three whole body mouse fetus sagittal sections (12 µm): One exposed to vehicle control with normal brain development, one exposed to valproic acid (500 mg/kg BW) with no visible malformations, and one exposed to valproic acid with an exencephaly NTD. Lipids shown were identified as discriminating lipid peaks using SCiLS ROC analysis (AUC < 0.2 or AUC > 0.8) for **(A)** whole brains and **(B)** spinal cords. Each dot represents the signal intensity at a single pixel in the MALDI image, with red dots representing outliers (1.5X interquartile range).


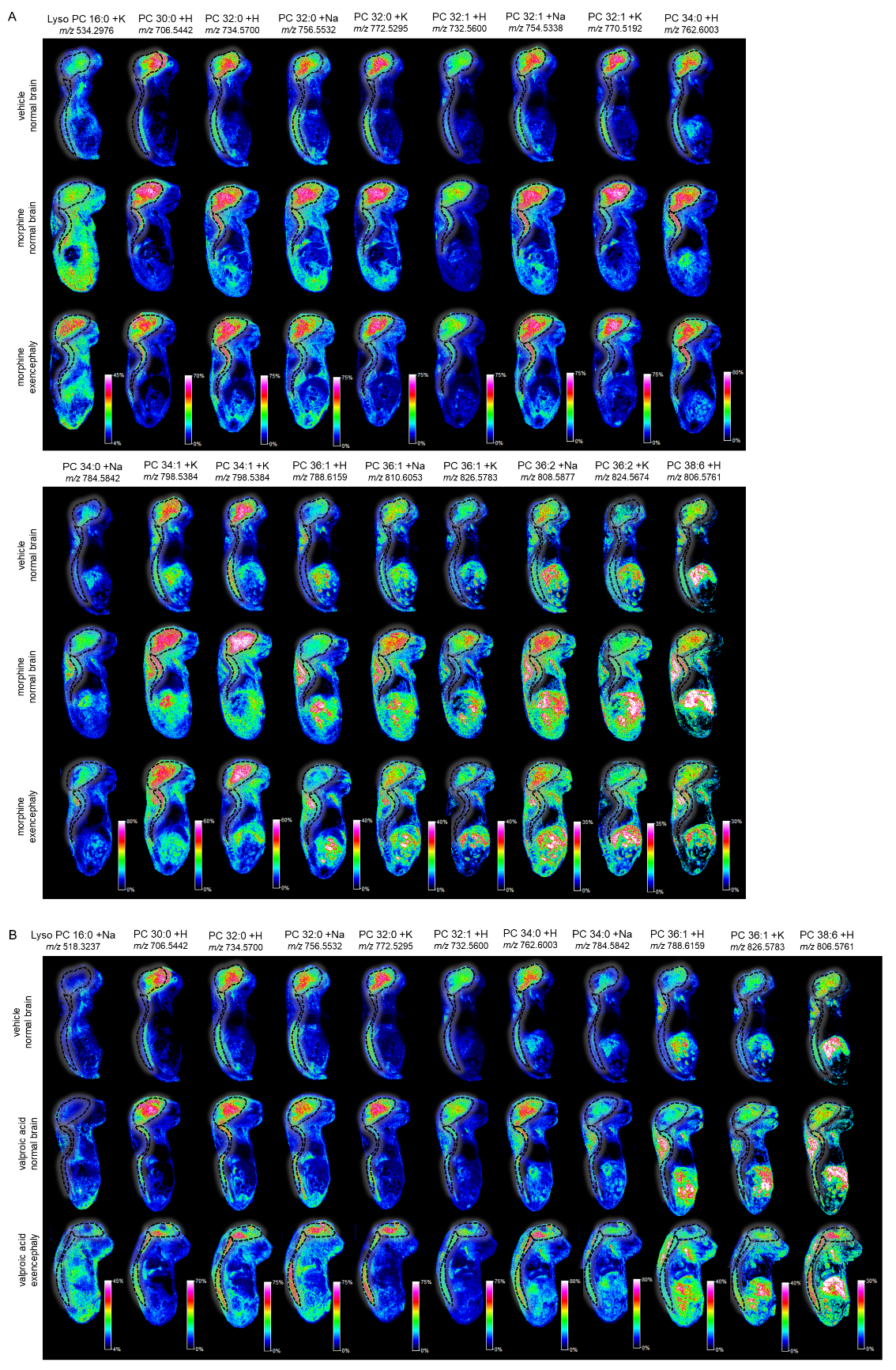


**Figure S3. Non-discriminating lipid peak intensities for MALDI scans of sagittal fetal sections.** Sagittal sections (12 µm) were taken from whole body mouse fetuses for MALDI imaging. Though SCiLS ROC analysis identified some targeted lipids as discriminating between vehicle and treatment groups for morphine-exposed (Figure 2) and VPA-exposed (Figure 3) fetuses, other targeted lipids were not discriminating. FlexImaging heatmaps show distributions and relative level intensities for the non-discriminating lipids normalized by total ion count for **(A)** morphine exposed mouse fetuses and **(B)** VPA-exposed mouse fetuses. Dotted lines outline the brain and spinal cord.


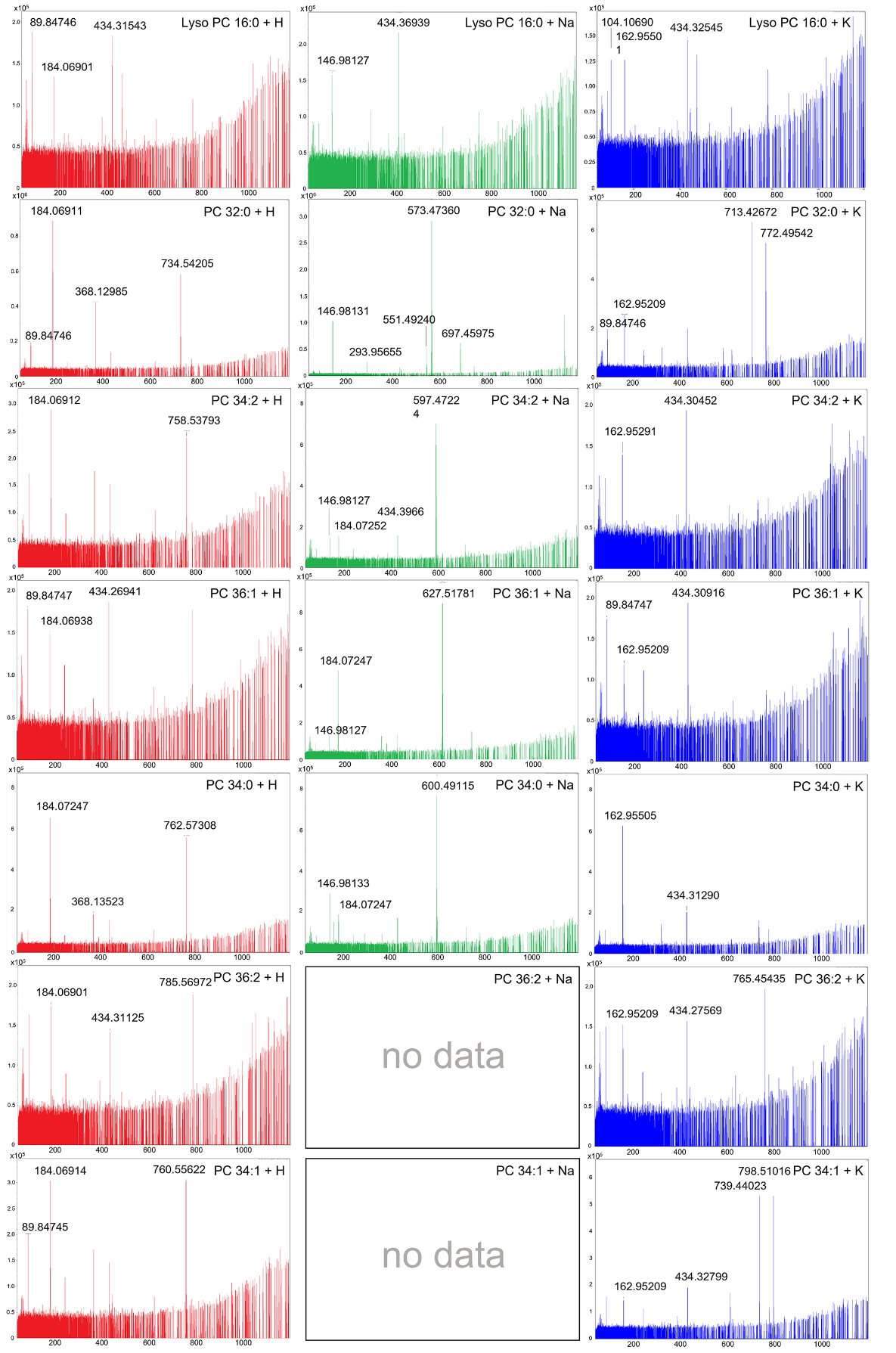


**Figure S4. CID spectrums of PC lipids.** CID spectrums were collected for the protonated (red), sodiated (green), and potassiated (blue) adducts of the lipids.


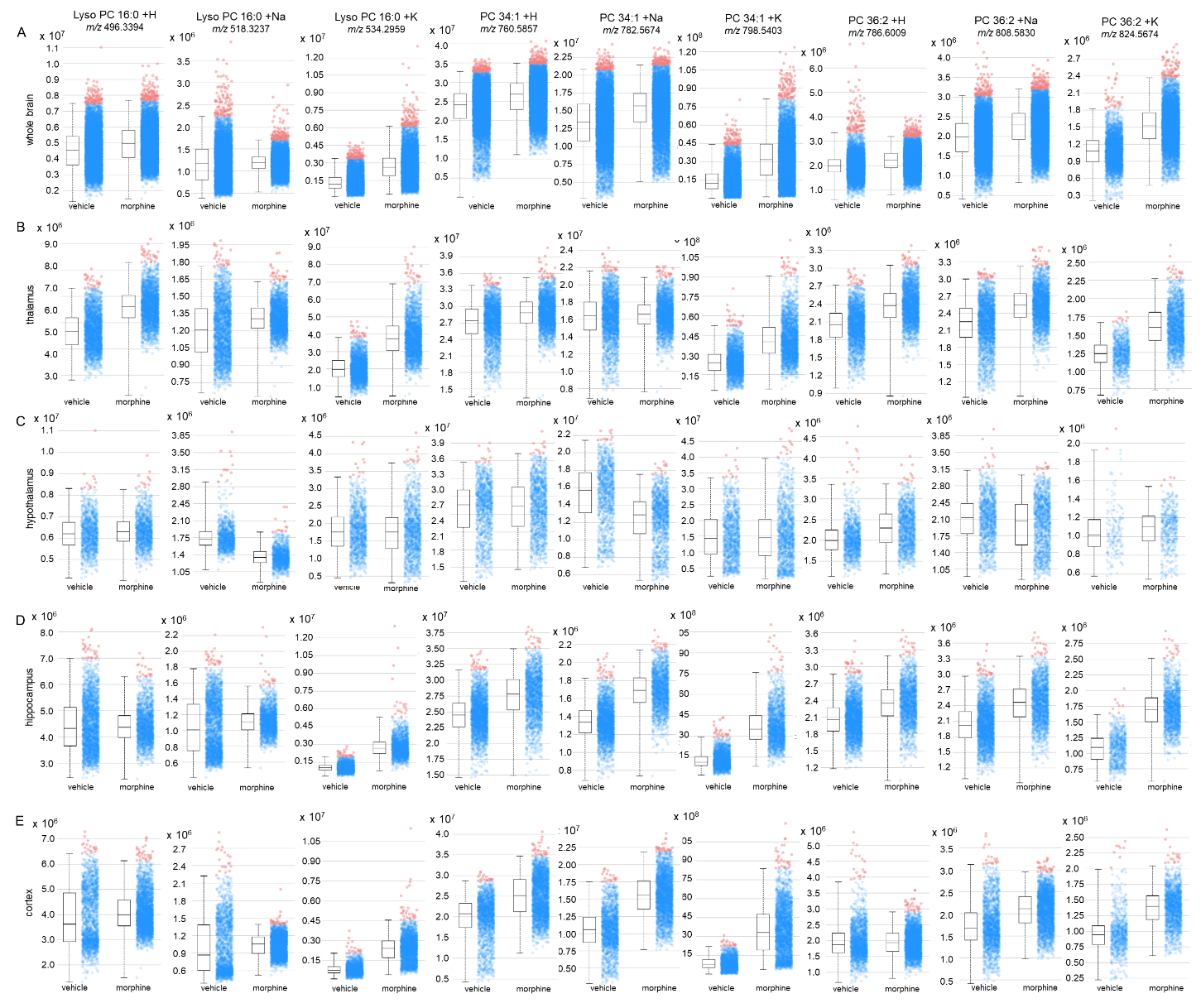


**Figure S5.** **Regional lipid levels in horizontal sections of morphine-exposed mouse fetal brains.** Horizontal sections (12 µm) were taken from mouse fetuses with normal brain development exposed to vehicle or morphine at 400 mg/kg BW for high resolution MALDI imaging of the right half of the brain (Figure 4). The regions for section collection were measured at around 6.5 mm from the end of the fetuses’ noses. SCiLS ROC analysis identified lyso PC 16:0, PC 34:1, and PC 36:2 as discriminating lipids (AUC < 0.2 or AUC > 0.8) for one or more brain region (Table 2). Boxplots show hydrogen, potassium, and sodium adducts for each discriminating lipid group in **(A)** the whole brain, **(B)** the thalamus **(C)** hypothalamus, **(D)** the cerebral cortex, and **(E)** the hippocampus. Each dot represents the signal intensity at a single pixel in the MALDI image, with red dots representing outliers (1.5 x interquartile range).

**Table S1. Top 200 peak results^a^ for mouse fetal brain sagittal sections**

| m/z | interval width (+/- Da) | maximum peak intensity^b^ | name |
| --- | --- | --- | --- |
| 110.77 | 0.003323 | 11648.98828 |  |
| 110.77 | 0.003323 | 11648.98828 |  |
| 110.78 | 0.003323 | 11648.98828 |  |
| 132.93 | 0.003988 | 17009.98047 |  |
| 132.93 | 0.003988 | 17009.98047 |  |
| 132.93 | 0.003988 | 17009.98047 |  |
| 132.93 | 0.003988 | 17009.98047 |  |
| 166.16 | 0.004985 | 10303.31055 |  |
| 166.16 | 0.004985 | 10303.31055 |  |
| 166.16 | 0.004985 | 10303.31055 |  |
| 177.01 | 0.005310 | 1859866.125 |  |
| 177.01 | 0.005310 | 1859866.125 |  |
| 177.01 | 0.005310 | 1859866.125 |  |
| 177.01 | 0.005311 | 1859866.125 |  |
| 177.01 | 0.005311 | 1859866.125 |  |
| 230.94 | 0.006928 | 2161842.5 |  |
| 230.95 | 0.006929 | 2161842.5 |  |
| 258.10 | 0.007743 | 196893.0938 |  |
| 258.11 | 0.007743 | 196893.0938 |  |
| 258.11 | 0.007743 | 196893.0938 |  |
| 273.02 | 0.008191 | 494086.9688 |  |
| 273.02 | 0.008191 | 1294483.875 |  |
| 273.02 | 0.008191 | 1294483.875 |  |
| 273.03 | 0.008191 | 16603147 |  |
| 273.03 | 0.008191 | 16603147 |  |
| 273.04 | 0.008191 | 16603147 |  |
| 273.05 | 0.008191 | 1302814.125 |  |
| 273.05 | 0.008192 | 1302814.125 |  |
| 274.03 | 0.008221 | 2476058.25 |  |
| 296.06 | 0.008882 | 394218 |  |
| 307.04 | 0.009211 | 1281033.625 |  |
| 313.03 | 0.009391 | 4008103.5 |  |
| 313.04 | 0.009391 | 4008103.5 |  |
| 317.04 | 0.009511 | 198192.6406 |  |
| 329.00 | 0.009870 | 3143231 |  |
| 329.01 | 0.009870 | 3143231 |  |
| 332.27 | 0.009968 | 9723.388672 |  |
| 332.28 | 0.009968 | 11826.67676 |  |
| 332.28 | 0.009969 | 14533.75391 |  |
| 332.29 | 0.009969 | 18253.97852 |  |
| 332.29 | 0.009969 | 25102.66992 |  |
| 332.30 | 0.009969 | 38206.84375 |  |
| 332.31 | 0.009969 | 113066.8438 |  |
| 332.31 | 0.009969 | 252354.5469 |  |
| 332.32 | 0.009969 | 252354.5469 |  |
| 332.32 | 0.009970 | 3797233.75 |  |
| 332.33 | 0.009970 | 3797233.75 |  |
| 332.34 | 0.009970 | 3797233.75 |  |
| 332.34 | 0.009970 | 261718.7969 |  |
| 332.35 | 0.009970 | 261718.7969 |  |
| 332.35 | 0.009971 | 129058.4688 |  |
| 332.36 | 0.009971 | 73183.21094 |  |
| 332.36 | 0.009971 | 47577.28906 |  |
| 332.37 | 0.009971 | 23876.31055 |  |
| 332.37 | 0.009971 | 18134.06445 |  |
| 332.37 | 0.009971 | 18134.06445 |  |
| 332.38 | 0.009971 | 13912.30664 |  |
| 332.38 | 0.009972 | 10842.35449 |  |
| 333.35 | 0.010001 | 35482.07813 |  |
| 347.01 | 0.010410 | 326169.125 |  |
| 347.02 | 0.010410 | 326169.125 |  |
| 348.06 | 0.010442 | 4288221 |  |
| 348.07 | 0.010442 | 4318167.5 |  |
| 348.08 | 0.010442 | 4318167.5 |  |
| 348.18 | 0.010445 | 5197479 |  |
| 348.19 | 0.010446 | 5197479 |  |
| 348.20 | 0.010446 | 5197479 |  |
| 348.20 | 0.010446 | 375260.0625 |  |
| 350.99 | 0.010530 | 928168.9375 |  |
| 360.36 | 0.010811 | 11449.48242 |  |
| 361.01 | 0.010830 | 93910.14063 |  |
| 364.07 | 0.010922 | 1113191.875 |  |
| 364.08 | 0.010922 | 833474.25 |  |
| 365.02 | 0.010951 | 204249.3594 |  |
| 366.96 | 0.011009 | 1339529.625 |  |
| 366.97 | 0.011009 | 502443.8438 |  |
| 369.35 | 0.011081 | 809727.625 |  |
| 372.31 | 0.011169 | 186739.0469 |  |
| 375.01 | 0.011250 | 375118.0625 |  |
| 376.98 | 0.011310 | 562754.5 |  |
| 386.03 | 0.011581 | 610757.1875 |  |
| 387.01 | 0.011610 | 991885.9375 |  |
| 390.98 | 0.011729 | 1047521.688 |  |
| 392.96 | 0.011789 | 1129787.625 |  |
| 393.30 | 0.011799 | 42177.1875 |  |
| 397.21 | 0.011916 | 1109394.125 |  |
| 398.33 | 0.011950 | 306433.5 |  |
| 399.13 | 0.011974 | 3050783.75 |  |
| 399.14 | 0.011974 | 7273754.5 |  |
| 399.16 | 0.011975 | 1689148.25 |  |
| 400.34 | 0.012010 | 613514.9375 |  |
| 402.02 | 0.012061 | 1258602.875 |  |
| 406.95 | 0.012209 | 1646828.5 |  |
| 408.01 | 0.012240 | 904904.125 |  |
| 409.04 | 0.012271 | 883812.4375 |  |
| 409.06 | 0.012272 | 1908960.125 |  |
| 409.07 | 0.012272 | 115917.7734 |  |
| 409.27 | 0.012278 | 53111.26172 |  |
| 423.98 | 0.012719 | 831325.125 |  |
| 424.34 | 0.012730 | 96928.03906 |  |
| 426.36 | 0.012791 | 639879.8125 |  |
| 426.37 | 0.012791 | 128156 |  |
| 428.04 | 0.012841 | 2757114.75 |  |
| 439.07 | 0.013172 | 273908.4688 |  |
| 449.36 | 0.013481 | 10167.94629 |  |
| 465.33 | 0.013960 | 8062.972168 |  |
| 465.99 | 0.013980 | 935427.3125 |  |
| 475.17 | 0.014255 | 10359.42676 |  |
| 483.02 | 0.014491 | 231245.375 |  |
| 487.97 | 0.014639 | 940950 |  |
| 496.34 | 0.014890 | 3089294.25 | Lyso PC 16:0 + H |
| 496.36 | 0.014891 | 215626.2656 |  |
| 501.01 | 0.015030 | 207455.25 |  |
| 501.03 | 0.015031 | 2209216.25 |  |
| 501.06 | 0.015032 | 143248.6094 |  |
| 503.95 | 0.015118 | 903083.125 |  |
| 508.00 | 0.015240 | 540960.25 |  |
| 518.32 | 0.015550 | 1021341.75 | Lyso PC 16:0 + Na |
| 522.36 | 0.015671 | 1011763.375 |  |
| 523.02 | 0.015690 | 412926.5313 |  |
| 524.37 | 0.015731 | 720013.5625 |  |
| 527.07 | 0.015812 | 768551.125 |  |
| 534.30 | 0.016029 | 1475059.375 | Lyso PC 16:0 + K |
| 542.07 | 0.016262 | 584850.5 |  |
| 576.24 | 0.017287 | 143771.6563 |  |
| 592.21 | 0.017766 | 163384.7188 |  |
| 607.26 | 0.018218 | 1913203.5 |  |
| 609.23 | 0.018277 | 1538724.875 |  |
| 609.25 | 0.018277 | 1538724.875 |  |
| 609.27 | 0.018278 | 22832434 |  |
| 609.28 | 0.018278 | 22832434 |  |
| 609.31 | 0.018279 | 1530656.25 |  |
| 609.31 | 0.018279 | 1530656.25 |  |
| 609.33 | 0.018280 | 1530656.25 |  |
| 609.35 | 0.018280 | 648388.4375 |  |
| 616.18 | 0.018485 | 1283948.375 |  |
| 617.18 | 0.018515 | 810880.375 |  |
| 637.04 | 0.019111 | 328987.0625 |  |
| 637.05 | 0.019112 | 328987.0625 |  |
| 650.43 | 0.019513 | 1247599.5 |  |
| 650.44 | 0.019513 | 1247599.5 |  |
| 664.12 | 0.019924 | 341219.5938 |  |
| 703.57 | 0.021107 | 1219027.875 |  |
| 703.58 | 0.021107 | 1219027.875 |  |
| 706.53 | 0.021196 | 897719.625 |  |
| 706.54 | 0.021196 | 897719.625 | PC 30:0 + H |
| 732.54 | 0.021976 | 2289546.75 |  |
| 732.56 | 0.021977 | 2289546.75 | PC 32:1 + H |
| 734.56 | 0.022037 | 5448103 |  |
| 734.57 | 0.022037 | 5448103 | PC 32:0 + H |
| 744.50 | 0.022335 | 574691.75 |  |
| 754.53 | 0.022636 | 1109162.125 | PC 32:1 + Na |
| 756.55 | 0.022696 | 2315587 | PC 32:0 + Na |
| 756.56 | 0.022697 | 2315587 |  |
| 758.56 | 0.022757 | 2021341.5 |  |
| 758.57 | 0.022757 | 2021341.5 |  |
| 760.58 | 0.022817 | 7510214.5 |  |
| 760.59 | 0.022818 | 7510214.5 | PC 34:1 + H |
| 760.63 | 0.022819 | 444819.9063 |  |
| 760.64 | 0.022819 | 444819.9063 |  |
| 761.31 | 0.022839 | 5543.17334 |  |
| 762.60 | 0.022878 | 970290.313 | PC 34:0 + H |
| 770.52 | 0.023115 | 1428880.625 | PC 32:1 + K |
| 772.53 | 0.023116 | 1428880.625 | PC 32:0 + K |
| 772.52 | 0.023176 | 3692635 |  |
| 772.53 | 0.023176 | 3692635 |  |
| 773.53 | 0.023206 | 1465309.875 |  |
| 773.54 | 0.023206 | 1465309.875 |  |
| 782.55 | 0.023477 | 3703963.75 |  |
| 782.56 | 0.023477 | 3703963.75 |  |
| 782.57 | 0.023477 | 3703963.75 | PC 34:1 + Na |
| 783.56 | 0.023507 | 1755601.125 |  |
| 783.57 | 0.023507 | 1755601.125 |  |
| 783.59 | 0.023508 | 1755601.125 |  |
| 784.58 | 0.023537 | 968075 |  |
| 784.58 | 0.023538 | 968075 | PC 34:0 + Na |
| 786.59 | 0.023598 | 1406587.75 |  |
| 786.60 | 0.023598 | 1406587.75 |  |
| 788.62 | 0.023658 | 1348943.125 | PC 36:1 + H |
| 788.62 | 0.023659 | 1348943.125 |  |
| 789.62 | 0.023689 | 450178.2188 |  |
| 796.53 | 0.023896 | 1185106.75 | PC 34:2 + K |
| 796.54 | 0.023896 | 1185106.75 |  |
| 798.53 | 0.023956 | 5071129 |  |
| 798.54 | 0.023956 | 5071129 | PC 34:1 + K |
| 798.55 | 0.023956 | 5071129 |  |
| 799.55 | 0.023987 | 2045754.5 |  |
| 800.57 | 0.024017 | 745427.8125 |  |
| 806.58 | 0.024197 | 542184.125 | PC 38.6 + H |
| 806.58 | 0.024197 | 542184.125 |  |
| 808.16 | 0.024245 | 425448.5 |  |
| 808.57 | 0.024257 | 1007984.125 |  |
| 808.58 | 0.024257 | 1007984.125 |  |
| 810.60 | 0.024318 | 1029219.625 | PC 36:1 + Na |
| 810.61 | 0.024318 | 1029219.625 |  |
| 810.62 | 0.024319 | 1029219.625 |  |
| 824.56 | 0.024737 | 839254.75 |  |
| 824.56 | 0.024737 | 839254.75 |  |
| 826.56 | 0.024797 | 904611.5 |  |
| 826.57 | 0.024797 | 904611.5 | PC 36:1 + K |
| ^a^ Peak list created using SCiLS feature finding tool over all sagittal section scanned regions.  ^b^ Peak data normalized by total ion count (TIC) in SCiLS. | | |  |

**Table S2. ROC analysis^a^ results for lipid adduct peaks in MALDI scans of morphine-exposed mouse fetal brain sagittal sections**

| **lipids** | | | | | **normal brain** | | | **exencephaly** | | | |
| --- | --- | --- | --- | --- | --- | --- | --- | --- | --- | --- | --- |
| **name** | **adducts** | | | ***m/z*** | **brain** | **spinal cord** | | **brain** | | **spinal cord** | |
| Lyso PC 16:0 | | + H | 496.3394 | | *0.088 ^b^* | | *0.077 ^b^* | | *0.065 ^b^* | | 0.212 |
|  |  | + Na | 518.3219 | | *0.168 ^b^* | | 0.502 | | *0.129 ^b^* | | 0.774 |
|  |  | + K | 534.2959 | | 0.236 | | 0.311 | | *0.067 ^b^* | | 0.618 |
| PC 30:0 | | + H | 706.5442 | | 0.326 | | 0.373 | | 0.564 | | 0.371 |
| PC 32:0 | | + H | 734.5700 | | 0.288 | | 0.364 | | 0.436 | | 0.353 |
|  | | + Na | 756.5508 | | 0.235 | | 0.532 | | 0.424 | | 0.628 |
|  | | + K | 772.5250 | | 0.314 | | 0.322 | | 0.349 | | 0.306 |
| PC 32:1 | | + H | 732.5600 | | 0.275 | | 0.487 | | 0.364 | | 0.587 |
|  | | + Na | 754.5338 | | 0.217 | | 0.315 | | 0.391 | | 0.312 |
|  | | + K | 770.5193 | | 0.311 | | 0.295 | | 0.390 | | 0.318 |
| PC 34:0 | | + H | 762.6003 | | 0.270 | | 0.613 | | 0.500 | | 0.688 |
|  | | + Na | 784.5829 | | 0.211 | | 0.570 | | 0.432 | | 0.645 |
| PC 34:1 | | + H | 760.5857 | | 0.227 | | 0.413 | | 0.386 | | 0.527 |
|  | | + Na | 782.5668 | | *0.188 ^b^* | | *0.137 ^b^* | | 0.312 | | *0.195 ^b^* |
|  | | + K | 798.5403 | | 0.280 | | 0.300 | | 0.362 | | 0.372 |
| PC 34:2 | | + K | 796.5254 | | 0.289 | | *0.171 ^b^* | | 0.529 | | 0.240 |
| PC 36:1 | | + H | 788.6159 | | 0.289 | | 0.521 | | 0.470 | | 0.785 |
|  | | + Na | 810.5988 | | 0.206 | | 0.342 | | 0.499 | | 0.337 |
|  | | + K | 826.5718 | | 0.235 | | 0.560 | | 0.461 | | 0.675 |
| PC 36:2 | | + H | 786.6009 | | *0.188 ^b^* | | 0.310 | | 0.399 | | 0.361 |
|  | | + Na | 808.5830 | | 0.251 | | 0.611 | | 0.424 | | 0.785 |
|  | | + K | 824.5565 | | 0.299 | | 0.292 | | 0.457 | | 0.374 |
| PC 38:6 | | + H | 806.5766 | | 0.307 | | 0.546 | | 0.516 | | 0.775 |

^a^ ROC analysis conducted using SCiLS discriminating features tool for control (group 1) versus morphine-exposed (group 2) fetal regions.

^b^ Italicized values indicate discriminating features (AUC < 0.2 or AUC > 0.8).

**Table S3. ROC analysis^a^ results for lipid adduct peaks in MALDI scans of VPA-exposed mouse fetal brain sagittal sections**

| **lipids** | | | | | **normal brain** | | | **exencephaly** | | | |
| --- | --- | --- | --- | --- | --- | --- | --- | --- | --- | --- | --- |
| **name** | **adducts** | | | ***m/z*** | **brain** | **spinal cord** | | **brain** | | **spinal cord** | |
| Lyso PC 16:0 | | + H | 496.3394 | | 0.354 | | 0.329 | | 0.270 | | *0.102 ^b^* |
|  |  | + Na | 518.3219 | | 0.390 | | 0.726 | | 0.227 | | 0.250 |
|  |  | + K | 534.2959 | | 0.405 | | 0.685 | | *0.096 ^b^* | | *0.147 ^b^* |
| PC 30:0 | | + H | 706.5442 | | 0.502 | | 0.368 | | 0.726 | | 0.260 |
| PC 32:0 | | + H | 734.5700 | | 0.512 | | 0.305 | | 0.595 | | 0.206 |
|  | | + Na | 756.5508 | | 0.472 | | 0.449 | | 0.313 | | 0.220 |
|  | | + K | 772.5250 | | 0.476 | | 0.349 | | 0.562 | | 0.213 |
| PC 32:1 | | + H | 732.5600 | | 0.387 | | 0.489 | | 0.272 | | 0.225 |
|  | | + Na | 754.5338 | | 0.360 | | 0.296 | | 0.517 | | *0.174 ^b^* |
|  | | + K | 770.5193 | | 0.383 | | 0.256 | | 0.460 | | *0.142 ^b^* |
| PC 34:0 | | + H | 762.6003 | | 0.473 | | 0.490 | | 0.468 | | 0.303 |
|  | | + Na | 784.5829 | | 0.424 | | 0.532 | | 0.443 | | 0.296 |
| PC 34:1 | | + H | 760.5857 | | 0.386 | | 0.385 | | 0.224 | | *0.159 ^b^* |
|  | | + Na | 782.5668 | | 0.379 | | *0.139 ^b^* | | 0.280 | | *0.043^b^* |
|  | | + K | 798.5403 | | 0.387 | | 0.280 | | 0.240 | | *0.121 ^b^* |
| PC 34:2 | | + K | 796.5254 | | 0.369 | | *0.170 ^b^* | | 0.453 | | *0.082 ^b^* |
| PC 36:1 | | + H | 788.6159 | | 0.440 | | 0.489 | | 0.304 | | 0.258 |
|  | | + Na | 810.5988 | | 0.433 | | 0.299 | | 0.337 | | *0.167 ^b^* |
|  | | + K | 826.5718 | | 0.406 | | 0.461 | | 0.331 | | 0.235 |
| PC 36:2 | | + H | 786.6009 | | 0.294 | | 0.232 | | 0.234 | | *0.069 ^b^* |
|  | | + Na | 808.5830 | | 0.397 | | 0.456 | | *0.185 ^b^* | | 0.220 |
|  | | + K | 824.5565 | | 0.296 | | 0.282 | | 0.296 | | *0.104 ^b^* |
| PC 38:6 | | + H | 806.5766 | | 0.478 | | 0.477 | | 0.312 | | 0.202 |

^a^ ROC analysis conducted using SCiLS discriminating features tool in control (group 1) versus VPA-exposed (group 2) fetal regions.

^b^ Italicized values indicate discriminating features (AUC < 0.2 or AUC > 0.8).
